# Age-Dependent Pre-Vaccination Immunity Affects the Immunogenicity of Varicella Zoster Vaccination in Middle-aged Adults

**DOI:** 10.3389/fimmu.2018.00046

**Published:** 2018-01-23

**Authors:** Marieke van der Heiden, Lia G. H. de Rond, Menno C. van Zelm, Guy A. M. Berbers, Annemieke M. H. Boots, Anne-Marie Buisman

**Affiliations:** ^1^Centre for Infectious Disease Control (Cib), National Institute for Public Health and the Environment (RIVM), Bilthoven, Netherlands; ^2^Department of Rheumatology and Clinical Immunology, University of Groningen, University Medical Centre Groningen, Groningen, Netherlands; ^3^Department of Immunology, Erasmus MC, Rotterdam, Netherlands; ^4^Department of Immunology and Pathology, Central Clinical School, Monash University and Alfred Hospital, Melbourne, VIC, Australia

**Keywords:** middle-aged adults, varicella zoster virus, T-cells, vaccination, cytokines, preexisting immunity

## Abstract

**Background:**

Prevention of infectious diseases is of high priority in the rapidly aging population. Unfortunately, vaccine responses in the elderly are frequently diminished. Timely vaccination of middle-aged adults might improve the immune responses to vaccines, although knowledge on pathogen-specific immune responses and factors affecting these responses, in middle-aged adults is currently limited. We thus investigated the immune responses after vaccination with Zostavax consisting of live-attenuated varicella zoster virus (VZV).

**Methods:**

Blood samples were taken pre-, 14 days, 28 days, and 1 year after a primary VZV vaccination (Zostavax) at middle age (*N* = 53, 50–65 years of age). VZV-specific IFNγ-producing cells were measured by ELISpot, activated T-cells by flow cytometry, antibody levels and cytokine responses by fluorescent bead-based multiplex immunoassays, and whole blood cellular kinetics by TruCOUNT analysis.

**Results:**

Robust short-term enhancement of the VZV-specific IFNγ-producing cell numbers was observed post-vaccination in the middle-aged adults. Remarkably, long-term enhancement of VZV-specific IFNγ-producing cell numbers was induced only in participants with low numbers of VZV-specific pre-vaccination IFNγ-producing cells, who were significantly older. These participants also showed enhancement of VZV-specific activated CD4 T-cells, contrary to “exhausted” VZV-specific CD8 T-cells in participants with high numbers of VZV-specific pre-vaccination IFNγ-producing cells. Finally, a high CD4/CD8 T-cell ratio was associated with low numbers of pre-vaccination VZV-specific IFNγ-producing cells.

**Conclusion:**

These results suggest that adults in their early sixties, who showed a high CD4/CD8 T-cell ratio and low numbers of VZV-specific IFNγ-producing cells, benefit from VZV vaccination. This provides important knowledge on factors affecting VZV-specific immune responses in middle-aged adults as well as for strategies to strengthen immunity before reaching old age.

## Introduction

Prevention of infectious diseases in the elderly is of high priority to establish healthy aging in the rapidly aging population. Unfortunately, vaccine effectivity in the elderly is low, leaving part of the elderly vulnerable for infections. Vaccination of middle-aged adults, before reaching old age, may be an alternative option to strengthen the memory immunity of the elderly. Currently, knowledge on pathogen-specific immune responses in middle-aged adults and factors affecting these immune responses is limited.

Herpes zoster is an infectious disease with a large disease burden in the elderly. Yearly, approximately 3–5/1,000 persons develop herpes zoster, also known as Shingles, of which the vast majority is of elderly age (1). Herpes zoster is caused by reactivation of the latent varicella zoster virus (VZV), causing chickenpox after the first encounter during childhood (2). The disease is characterized by painful rashes, mostly located at a single dermatome, which may develop into long-lasting complications such as post herpetic neuralgia (2, 3). Advancing age and immune suppression are known as the strongest risk factors for herpes zoster, with a sharp increase in cases seen after the age of 50 (3–5). Due to rapid aging of the world population, the incidence of herpes zoster is likely to increase (1, 6–8). Consequently, understanding the VZV-specific immune responses and factors affecting this immunity is crucial to decrease the herpes zoster disease burden.

Varicella zoster virus-specific cell-mediated immunity (CMI) is essential in the protection against virus reactivation (9). Particularly, VZV-specific IFNγ-producing T-cells are seen as the best surrogate marker for protection against herpes zoster, although the exact number of protective cells remains to be established (10, 11). A decline in VZV-specific CMI with age has been linked to increased susceptibility to virus reactivation and subsequent herpes zoster disease (12–15). By contrast, the role of VZV-specific antibodies in the protection against herpes zoster is controversial, because contrary to CMI, VZV-specific antibody concentrations do not decrease with advancing age (13, 16). Nevertheless, some studies suggest a positive correlation between antibody levels and protection against herpes zoster (10, 17).

In 2006, a live-attenuated vaccine, Zostavax, was licensed to prevent herpes zoster disease in persons of 50 years and older (18). However, due to immunological aging, vaccine efficacy strongly decreases with advancing age (10, 12, 19). The low vaccine efficacy at old (>65 years) age is one of the main reasons for a fierce debate about the implementation of the Zostavax vaccine in national vaccination programs. Consequently, solutions are warranted to decrease the herpes zoster disease burden in the elderly. Currently, a herpes zoster subunit vaccine containing VZV glycoprotein E and the AS01_B_ adjuvant system is under regulatory evaluation after showing promising results in prevention of herpes zoster in the elderly (20–22). Yet, knowledge on factors influencing vaccine-induced VZV-specific immune responses during aging is lacking.

To improve our understanding of VZV-specific immune responses, we investigated the cellular and humoral immune responses after VZV (Zostavax) vaccination in middle-aged adults (50–65 years of age), an interesting target group for harnessing memory immunity before reaching old age. Moreover, we studied factors associated with vaccine immunogenicity in these middle-aged adults to be able to better predict the vaccine responses.

## Materials and Methods

### Study Design and Participants

Fifty-three middle-aged adults (50–65 years of age) were included in this phase IV single-center and open-label study. Participants were recruited from an urban area in the middle of the Netherlands in March 2015. Potential participants were excluded based on the following criteria: antibiotic use or fever (>38°C) within the last 14 days, diseases demanding immune suppressive treatment within the last 3 months, a known or suspected immune deficiency, a blood coagulation disorder, a neurologic disorder, administration of blood products in the past 6 months, serious surgery within the last 3 months, the use of hormone supplementation, a suspected allergy toward the vaccine components, a history of serious adverse events after previous vaccinations, a previous varicella zoster episode, a previous Zostavax vaccination, and any vaccination in the month before enrollment. Written informed consent was obtained from all participants before the study. All procedures were in accordance with the Declaration of Helsinki. The study was approved by the Medical Research Ethics Committees United (Mec-U) in Nieuwegein, the Netherlands (NTR4636).

### Vaccination and Blood Sampling

**Figure 1:**
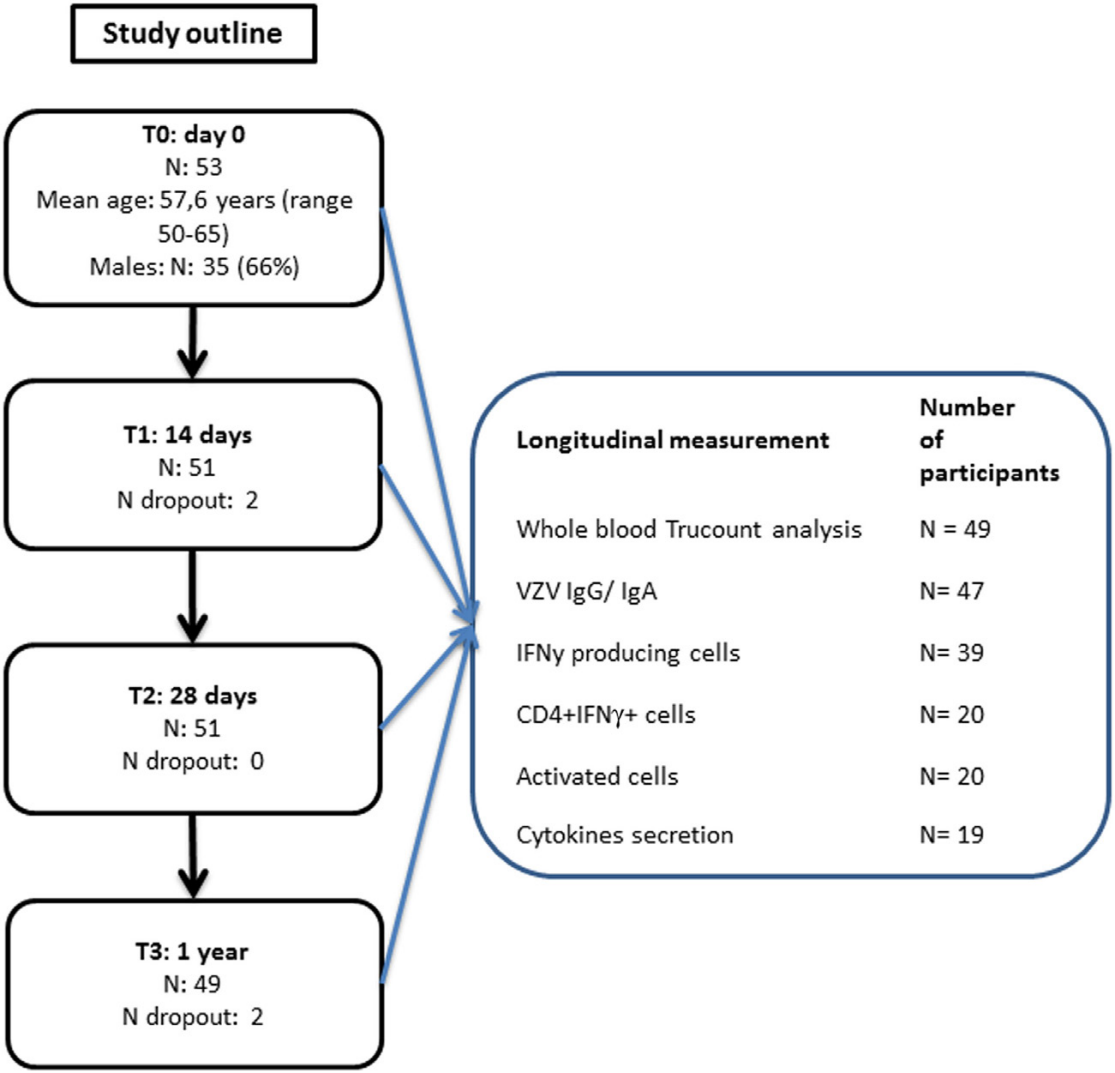
Participants flowchart.

All participants received a single dose of the live-attenuated varicella zoster vaccination (Zostavax; Sanofi Pasteur MSD), containing not less than 19,400 PFU. The vaccine was subcutaneously administered. A blood sample was taken from all participants before the vaccination. Post-vaccination, blood samples were taken after 14 days, 28 days, and 1 year (Figure 1). Peripheral blood mononuclear cells (PBMCs) were isolated at all time points using vacutainer cell preparation tubes (CPT) containing sodium citrate (BD Biosciences), according to the manufacturer’s instructions (23). Plasma samples were collected after initial spinning of the CPT tubes and were filtered using a Corning^®^Costar^®^ Spin^®^ X filter (Sigma-Aldrich) and were frozen at −20°C before further use. After the initial collection, the PBMCs were washed with RPMI-1640 medium (Gibco) supplemented with 1% heat inactivated fetal calf serum (FCS, Gibco) and 1% penicillin and streptomycin (Lonza). PBMCs were counted and frozen in a 90% FCS, 10% DMSO solution at −135°C until further use. Moreover, pre-vaccination, and at 14 and 28 days post-vaccination, an additional blood sample was collected in tubes containing lithium heparin (BD) for detailed cellular immune phenotyping. Both the vaccinations and blood samplings were performed in the evening hours.

### Whole Blood Immune Phenotyping

The whole blood cellular immune phenotyping was performed within 18 h after blood collection. Pre-vaccination, the absolute numbers of lymphocytes, monocytes, granulocytes, CD3+ T-cells, NK cells, B-cells, and the different B-cell subsets (naïve mature, transitional, natural effector, memory B-cells, and plasma cells) were determined with a lyse no-wash protocol using TruCOUNT tubes (BD). The following antibodies were used: CD3(UCHT1)-BV711, CD16(B73.1)-PE, and CD38(HB7)-APC-H7 (all from BD Biosciences), CD45(GA90)-OC515 and CD56(C5.9)-PE (both from Cytognos, Salamanca, Spain), CD27(M-T271)-BV421 and IgD(IA6-2)-FITC (both from BioLegend, San Diego, CA, USA), and CD19(J3-119)-PE-Cy7 (Beckman Coulter, Fullerton, CA, USA). Moreover, a detailed immune phenotyping was performed separately on the whole blood pre-vaccination samples using: CD4(RPA-T4)-BV510, CD45RA(HI100)-BV605, and CD28(CD28.2)-PerCP-Cy5.5 (all from BioLegend), CCR7(150503)-PE-CF594, CD8(SK1)-APC-H7, CD25(2A3)-FITC, TCRgd(11F2)-PE-Cy7, and CD127(hIL-7R-M21)-PE (all from BD Biosciences), and CXCR5(51505)-APC (R&D Systems, Minneapolis, MN, USA). Absolute numbers of T-cell subsets were calculated using the CD3+ T-cell numbers from the TruCOUNT analysis. In addition, 14 and 28 days post-vaccination whole blood TruCOUNT analysis was performed using the following antibodies: CD27(M-T271)-BV421, CD45RA(HI100)-BV605, IgD(IA6-2)-FITC, CD8(SK1)-FITC, CD4(OKT4)-PerCP-Cy5.5 (all BioLegend), CD3(UCHT1)-BV711, CD25(2A3)-PE, CCR7(150503)-PE-CF594, TCRgd(11F2)-PE-Cy7, and CD38(HB7)-APC-H7 (all BD Biosciences), CD45(GA90)-OC515 (Cytognos), CD19(J3-119)-PE-Cy7 (Beckman Coulter), and CXCR5(51505)-APC (R&D Systems). Gating strategies for the different cell subsets were applied as previously described (24). An overview of the different markers used to specify the different cell populations is given in Table S2 in Supplementary Material. Flow cytometric analyses were performed on a 4-laser LSR Fortessa (BD Biosciences) using standardized measurement settings (25), and data analysis using FacsDiva V8 (BD Biosciences) and FlowJo V10 (FlowJo Company, Ashland, OR, USA).

### VZV-Specific IFNγ and Granzyme B (GrzB) ELISpot

Multiscreen 96-well ELISpot plates (Merck Millipore) were shortly activated with 70% ethanol after which they were thoroughly washed with PBS (Tritium Microbiology) and either coated with antihuman IFNγ antibody 1-D1K or antihuman GrzB GB10 (1 mg/mL; Mabtech). The coated plates were kept overnight at 4°C. The next day, the plates were blocked with AIM-V medium (ThermoFisher) containing 5% human AB serum (Sigma-Aldrich), hereafter called T-cell medium, for at least 1 h at 37°C to avoid non-specific binding. The same batch of human AB serum was used for all samples to avoid differences in antigen concentration during T-cell stimulation. After a quick thawing of the PBMCs in T-cell medium, PBMCs were thoroughly washed and distributed in concentrations of 3 × 10^5^, 1.5 × 10^5^, and 7.5 × 10^4^ cells/well over the pre-coated IFNγ or GrzB plates. The PBMCs were stimulated with the mock (negative control), 6 µg/mL VZV-specific purified antigen (VZ10 strain; Genway), or 1 µg/mL *Staphylococcus aureus* enterotoxin B (SEB, positive control) (Sigma-Aldrich), again in a threefold dilution. All stimulations were performed in duplicate. All time points of one participant were tested on the same ELISpot plate, to reduce assay variation. The plates were incubated for 48 h at 37°C with 5% CO_2_. Next, supernatants of the stimulated cells were collected and frozen at −80°C. Subsequently, the plates were washed thoroughly and incubated for 2 h at 37°C with either mouse antihuman IFNγ antibody 7-B6-1 or mouse antihuman GrzB antibody GB11 (both Mabtech). After a washing step, a mixture containing Extravidin–alkaline (Sigma-Aldrich) was added for 1 h at room temperature. Finally, plates were washed and spots were developed by addition of a SIGMAFAST™ BCIP/NBT (Sigma-Aldrich) solution. After drying, plates were scanned with the Epson ELISpot Scanner, and the spots were counted with a standardized protocol using the AELVIS software. Numbers of VZV-specific IFNγ and GrzB producing cells are presented per 10^6^ PBMCs after subtraction of the spots in the mock control. Mock controls on average contained 17 spots/10^6^ PBMCs for IFNγ and 20 spots/10^6^ PBMCs for GrzB. An NK-cell depletion experiment (using CD56 magnetic bead separation) was performed to estimate the role of NK cells in the IFNγ production as measured in the ELISpot assays.

### VZV-Specific IgG and IgA

Varicella zoster virus-specific IgG concentrations (IU/mL) at the different time points were measured using a bead-based immunoassay as described previously (26). VZV IgA concentrations (NTU) were measured using an enzyme-linked immunoassay (Genway Biotech, Inc., San Diego, CA, USA) according to the manufacturer’s instructions.

### CMV Serology

CMV IgG concentrations were determined in the plasma samples using an enzyme-linked immunoassay (ETI-CYTOK-G Plus, P002033, DiaSorin, Saluggia, Italy) according to the manufacturer’s indications and as described earlier (24).

### VZV-Specific T Cell Activation and IFNγ Production

Peripheral blood mononuclear cells were thawed as before. Thereafter, 10^6^ cells/well were stimulated with mock (negative control) or 6 µg/mL VZV-specific purified antigen (VZ10 strain; Genway) in a 48-well plate. Moreover, 5 × 10^5^ cells/well were stimulated with 1 µg/mL SEB (positive control) (Sigma-Aldrich). The cells were incubated for 72 h at 37°C with 5% CO_2_. During the last 5 h GolgiPlug protein transport inhibitor containing Brefeldin A (1,000× dilution, BD) was added to each well. After a thorough washing, cells were incubated for 30 min with a mixture of Life-Death Zombie Aqua fluorescent dye (BioLegend) and surface antibodies in FACS buffer, containing PBS with 0.5% BSA and 2 mM EDTA. The following antibodies were used for surface staining: CD3(SK7)-PerCP, CD4(RPA-T4)-ACP, CD45RA(L48)-PE-Cy7, CCR7(3D12)-BV605, CD56(NCAM16.2)-BV711 (all BD), CD38(HIT2)-BV786 (BioLegend), CD8(CLBT8/4, H8)- FITC (Sanquin), and HLA-DR(LN3)-APCefluor780 (eBiosciences). Subsequently, cells were washed with PBS, and permeabilized for 20 min with cytofix/cytoperm (BD). A perm/wash solution (BD) was used to wash the cells. In addition, the cells were stained for 30 min with IFNγ (25723.11)-PE. After an additional washing step, the cells were resuspended in FACS buffer and immediately measured on a 4-laser LSR Fortessa (BD), and the data were analyzed using FlowJo V10. Frequencies of activated (CD38+ HLA-DR+) and CD4+ IFNγ+ (data not shown) VZV-specific cells were calculated after subtraction of the mock control.

### Cytokine Detection in Supernatants

After 48 h of PBMC stimulation, IL2, TNFα, IL5, IL13, and IL10 concentrations in the supernatants were determined as previously described (27). Samples with concentrations below the lower limit of quantification were assigned half the concentration of the lowest measurement. Cytokine concentrations in the mock controls were subtracted from those in the antigen stimulated samples.

### Statistics

Only data of participants with measurements at all different time points were used for analysis. The numbers of VZV-specific IFNγ-producing cells, cytokine concentrations, activated cells, and the whole blood TruCOUNT responses at all the different time points were compared with the Friedman test. Only if this test yielded significant outcomes, the Wilcoxon signed rank test was applied to determine significant differences between two separate time points analyzed. The VZV-specific IgG and IgA responses were log-transformed after which the ANOVA was used to compare the different time points. Corrections for multiple testing were used as indicated under the figures/tables. Participants with low and high pre-vaccination immunity were compared at all time points by using the Mann–Whitney *U* test. Correlations were determined by the Spearman’s rho correlation test. Geometric means with the 95% confidence intervals were indicated in the graphs. The boxplots used in the figures are plotted from the min to max values with indication of the median. The whole blood absolute cell numbers were compared at the different time points between the participants with low and high pre-vaccination CMI with the Mann–Whitney *U* test. The geometric means of these groups were normalized to *z*-scores using the geometric means and standard deviation of the total group to be presented in the heat maps. GraphPad V7 and SPSS V22.0 were used.

## Results

### Participant Characteristics

A total of 53 middle-aged adults participated in this study (mean age: 57.6; range 50–65; 66% male) (Figure 1). After a pre-vaccination blood drawing, all participants received the Zostavax vaccine. Follow-up blood samples were drawn at 14 days, 28 days, and 1 year post-vaccination. Finally, 49 (92.5%) of the participants completed the study. Additional participant health characteristics are presented in Table S1 in Supplementary Material. Due to sample availability, distinct numbers of participant samples were used for the various assays (Figure 1).

### Robust Short-term VZV-Specific Cellular and Humoral Vaccine Responses in Middle-aged Adults

**Figure 2:**
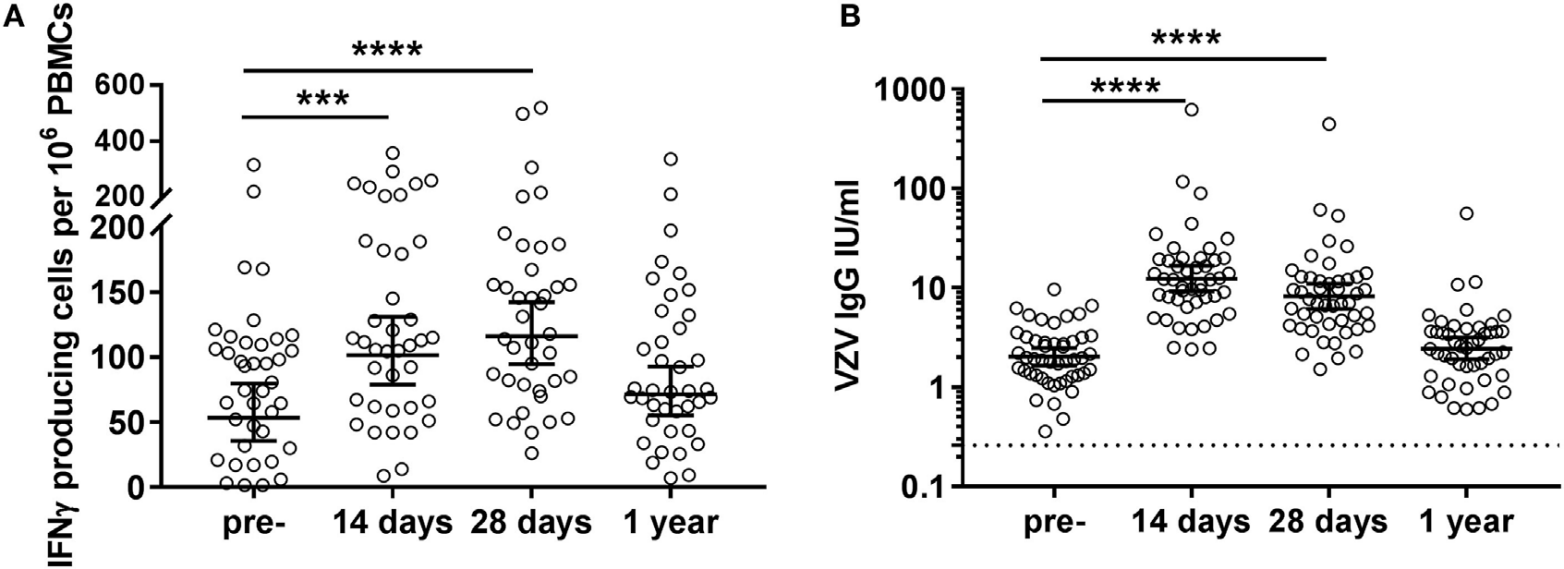
Varicella zoster virus (VZV)-specific cellular and humoral immunity post VZV vaccination in middle-aged adults. **(A)** VZV-specific IFNγ-producing cell responses per 10^6^ peripheral blood mononuclear cells (PBMCs), as measured by ELISpot, at the different time points pre- and post-vaccination (*N* = 39) after subtraction of the values in the mock control. The different time points were compared with the Wilcoxon singed rank test preceded by the Friedman test and corrected for multiple comparisons. **(B)** VZV-specific IgG responses at the different time points pre- and post-vaccination (*N* = 47). The IgG (IU/mL) concentrations were log-transformed to reach the normality distribution and compared with the ANOVA with correction for multiple testing. The dotted lines indicate the seropositivity thresholds. In all graphs, the geometric mean values with 95% confidence interval are indicated. *p < 0.05, ***p < 0.001, and ****p < 0.0001.

Pre-vaccination, highly variable numbers of VZV-specific IFNγ-producing cells per 10^6^ PBMCs were observed in the circulation of middle-aged adults (geometric mean 53.4 [35.8–79.6]) (Figure 2A). These cells significantly increased shortly after vaccination (14 days: geometric mean 101.6 [79.0–130.8]; 28 days: geometric mean 115.9 [94.5–142.3]) and returned to baseline values 1-year post-vaccination (geometric mean 71.5 [55.1–92.8]) (Figure 2A). Importantly, we observed a negative correlation between pre-vaccination VZV-specific IFNγ-producing cell numbers and the fold change of VZV-specific IFNγ-producing cells post-vaccination (14 days: rho: −0.669, 28 days: rho: −0.714, and 1 year: rho: −0.610; all *p* < 0.0001). We did not observe correlations between the cellular and humoral responses, indicating that these responses develop independently (data not shown). A preliminary NK cell depletion experiment indicated that the majority of this IFNγ was produced by the T-cells (data not shown).

All participants possessed pre-vaccination IgG antibody concentrations above the VZV seropositivity level of 0.26 IU/mL, as established by van Lier et al. (16) (geometric mean 2.0 [1.7–2.5]), indicative of previous infection with the VZV virus in all participants (Figure 2B). These VZV-specific IgG antibodies were found significantly increased at days 14 (geometric mean 12.4 [9.2–17.6]) and 28 (geometric mean 8.2 [6.1–11.1]) post-vaccination. Similar to the IFNγ T-cell responses, no significantly elevated IgG responses were observed 1-year post-vaccination (geometric mean 2.5 [1.9–3.1]) (Figure 2B).

### Long-term Enhancement of VZV-Specific IFNγ-Producing Cell Numbers in Participants with Low Pre-Vaccination CMI

**Figure 3:**
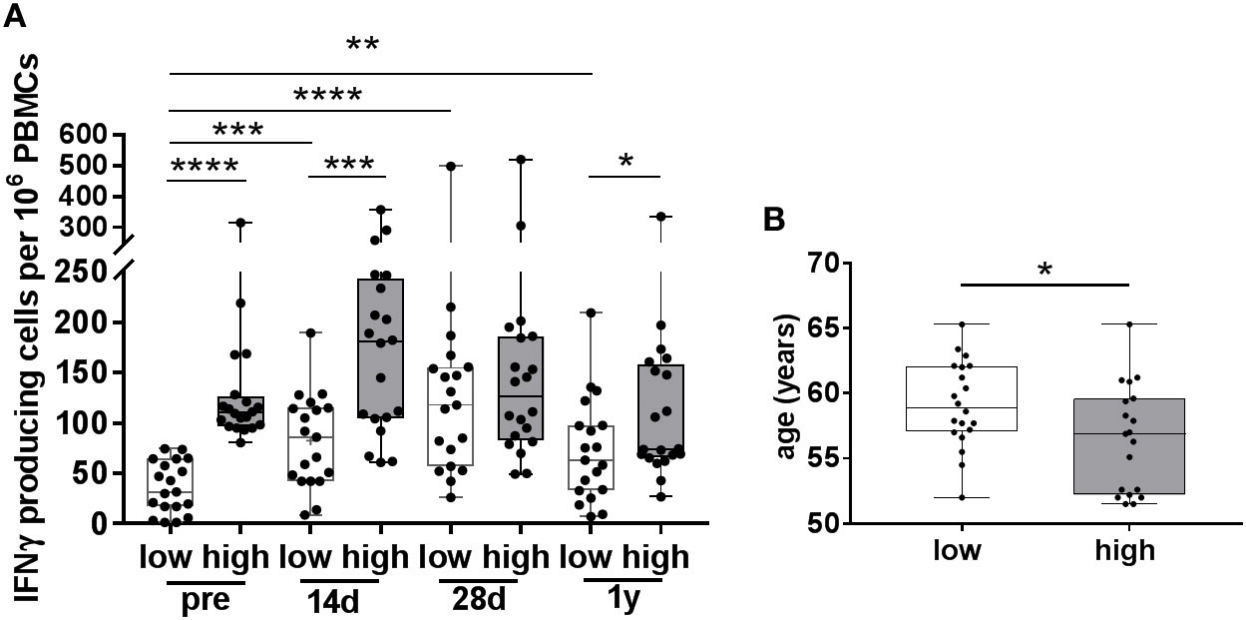
Varicella zoster virus (VZV)-specific responses in participants with low and high pre-vaccination IFNγ-producing cells. **(A)** The number of VZV-specific IFNγ-producing cells, as measured by ELISpot, in participants with low (white boxplots, *N* = 19) and high (gray boxplots, *N* = 20) pre-vaccination IFNγ-producing cells. These groups were determined using the median number of pre-vaccination IFNγ-producing cells. Participants in the low pre-vaccination group possessed numbers of VZV-specific IFNγ-producing cells below the median, compared with numbers above the median in the high pre-vaccination group. **(B)** The age distribution of participants with low and high pre-vaccination IFNγ-producing cells. All boxplots are plotted from the min to max values with indication of the median. The low and high responders were compared with the Mann–Whitney *U* test. The different time points were compared with the Wilcoxon signed rank test preceded by the Friedman Test with correction for multiple testing. *p < 0.05, **p < 0.01, ***p < 0.001, and ****p < 0.0001.

Since we observed a negative correlation between the numbers of pre-vaccination IFNγ-producing cells and the vaccine-induced increase in these cells, we determined whether differential vaccine responses were observed in participants with low and high numbers of pre-vaccination VZV-specific IFNγ-producing cells (Figure 3), hereafter called participants with low and high pre-CMI. The median number of pre-vaccination spots was determined, and participants below the median were considered to have low pre-CMI whereas participants above the median were considered to have high pre-CMI. Remarkably, only participants with low pre-CMI showed enhancement of the number of IFNγ-producing cells post-vaccination, which was maintained up until 1 year (fold change 14 days: 2.7, 28 days: 4.3, and 1 year: 2.2) (Figure 3A). On the contrary, numbers of IFNγ-producing cells were not significantly enhanced post-vaccination in participants with high pre-CMI (fold change 14 days: 1.3, 28 days: 1.1, and 1 year: 0.8) (Figure 3A). One-year post-vaccination, some of these participants even showed trends toward reduced numbers of IFNγ-producing cells compared with pre-vaccination. Interestingly, participants with low pre-CMI were significantly older than the participants with high pre-CMI, even within this small age-range (Figure 3B).

The two groups showed similar VZV-specific IgG concentrations (Figure S1A in Supplementary Material), confirming the independence of the VZV-specific IgG and CMI responses. Noteworthy, IgA responses significantly differed between the two groups, with stronger responses in the participants with low pre-CMI (Figure S1B in Supplementary Material).

To enlarge our understanding of the difference in T-cell responses between the participant with low and high pre-CMI, we compared the production of several cytokines in the cell culture supernatants after 48 h of culture. Significant differences were found between the two groups in VZV-specific Th1 and Th2 cytokines. Post-vaccination, significant enhancement of the VZV-specific IL2, TNFα, IL13, and IL5 secretion was observed in the low pre-CMI group, whereas no significant responses were found in the high pre-CMI group. Noteworthy, no significant IL10 and GrzB responses were observed in both groups (Figure S2 in Supplementary Material).

Overall, our results indicate strong VZV-specific cytokine and IgA responses in the circulation of middle-aged adults with low pre- VZV-specific CMI, whereas this response was absent in the circulation of adults with high pre-CMI. Importantly, a low pre-CMI was associated with higher age in this group.

### Differential Responses in VZV-Specific Activated (CD38+ HLA-DR+) T-Cells Post-Vaccination in Participants with Low and High Pre-CMI

**Figure 4:**
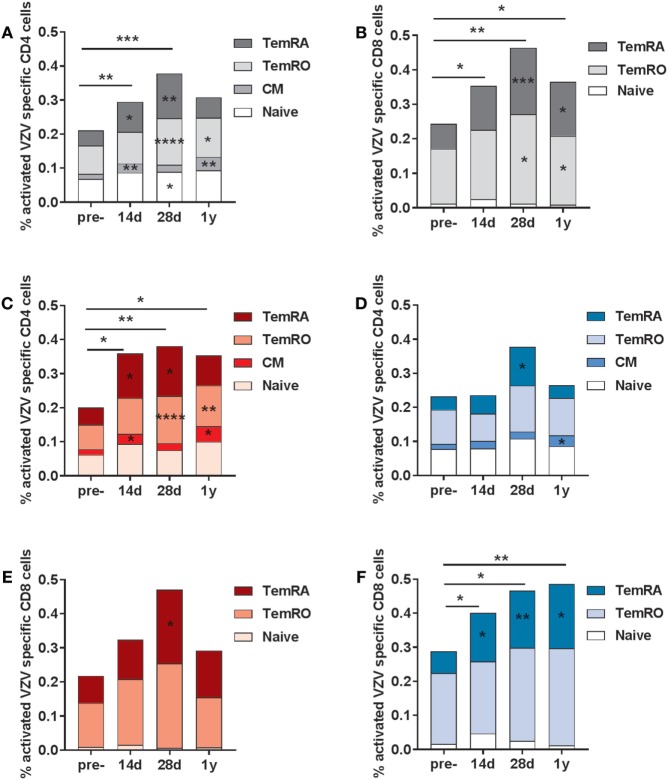
Varicella zoster virus (VZV)-specific activated (CD38+ HLA-DR+) T-cells. The VZV-specific frequency of activated T-cells (based on the double expression of CD38 and HLA-DR) in the CD4 **(A)** and CD8 **(B)** T-cell compartments in the total group of participants (*N* = 20). The VZV-specific activated CD4 T-cells in participants with low **(C)** and high **(D)** pre-cell-mediated immunity (CMI). The VZV-specific activated CD8 T-cells in participants with low **(E)** and high **(F)** pre-CMI. The geometric mean values were used in the graphs. The different time points were compared with the pre-vaccination frequencies with the Wilcoxon signed rank test preceded by the Friedman test. The stars in the bars indicate significant differences for specific subsets as compared with the pre-vaccination levels, whereas the starts above the bars indicate differences in the total CD4+ of CD8+ VZV-specific activated cells as compared with the pre-vaccination time point. *p < 0.05, **p < 0.01, ***p < 0.001, and ****p < 0.0001.

Subsequently, we investigated the frequencies of VZV-specific activated T-cells, both within the CD4 and CD8 T-cell compartment (gating strategy presented in Figure S3 in Supplementary Material). Activated cells were based on the double expression of HLA-DR and CD38 (gating strategy in Figure S4 in Supplementary Material). Activated VZV-specific CD8 CM cells could not be detected, due to the low frequencies of these cells in the circulation (28). We observed elevated frequencies of activated VZV-specific CD4 T-cells at 14 and 28 days post-vaccination, mainly consisting of CM, TemRO, and TemRA cells (Figure 4A). After 1 year, elevated frequencies of activated CD4 CM and TemRO cells were still found. The numbers of VZV-specific activated CD8 T-cells were significantly enhanced at all time points post-vaccination, and mainly consisted of TemRO and TemRA cells (Figure 4B). Remarkably, activated VZV-specific cell responses were significantly different between the participants with low and high pre-CMI (Figures 4C–F). Participants with low pre-CMI showed strong enhancement of the activated VZV-specific CD4 cells (CM, TemRO, and TemRA) and only a small enhancement of the CD8 TemRA response (Figures 4C,E). By contrast, participants with high pre-CMI showed a CD8 T-cell oriented response, with increased frequencies of VZV-specific CD8 TemRA cells up until 1 year (Figure 4F). However, 1-year post-vaccination, this group also showed some enhancement of the VZV-specific CD4 CM T-cells (Figure 4D). Overall, these responses suggest differential induction of T-cell responses in participants with low and high pre-CMI, with a more CD4 T-cell oriented response in participants with low pre-CMI as compared with a more CD8 TemRA oriented response in participants with high pre-CMI.

### Whole Blood Lymphocyte Subset Kinetics Mirror the Differential Vaccine Responses in Participants with Low and High Pre-CMI

**Figure 5:**
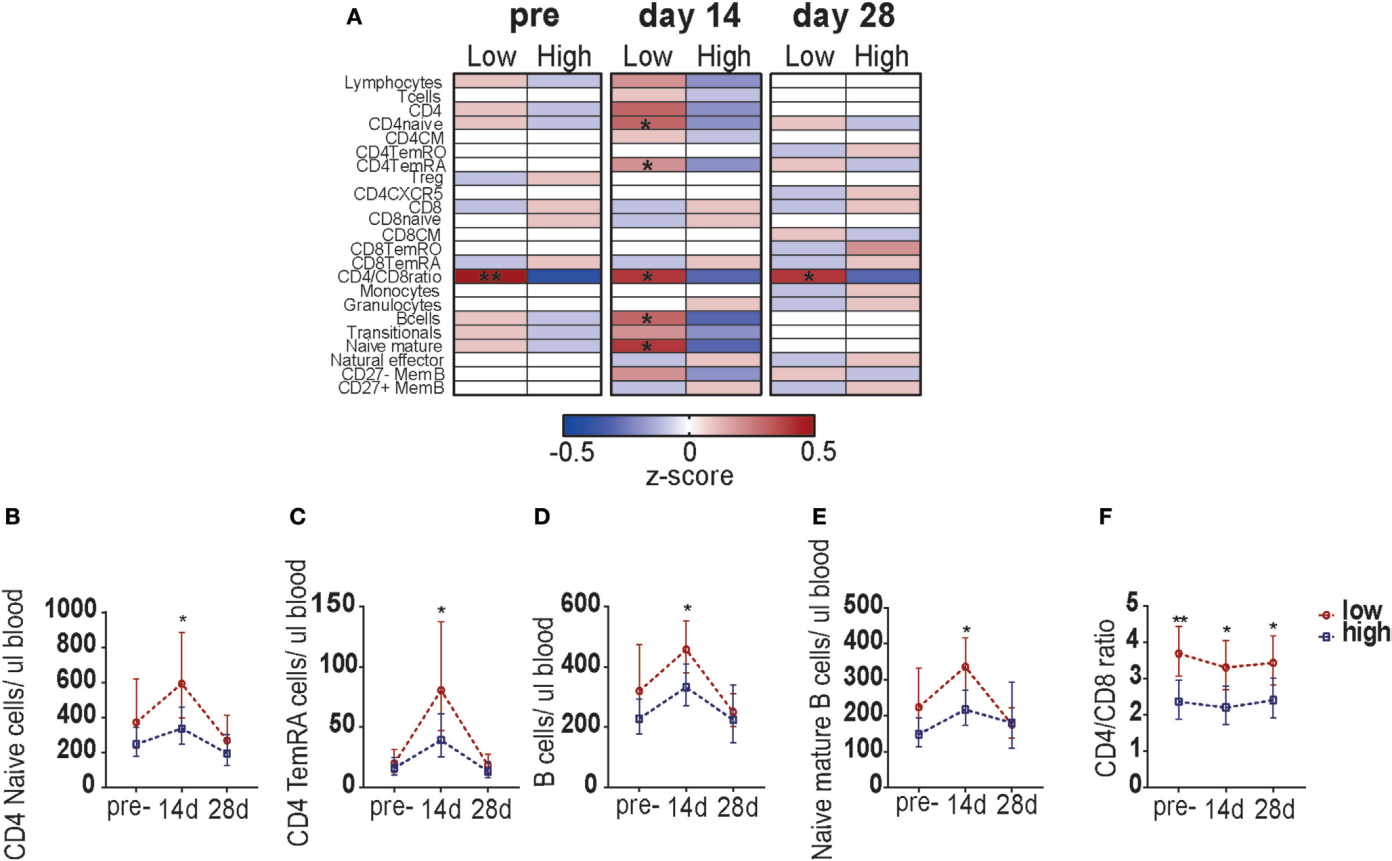
Comparison of leukocyte subset kinetics between adults with low or high pre-cell-mediated immunity (CMI). **(A)** Comparison of the whole blood absolute immune cell numbers between participants with low and high pre-CMI at the pre- as well as 14 and 28 days post-vaccination time points. The absolute cell numbers were normalized to *z*-scores, using the geometric means of the low and high group, respectively. *z*-Scores ranged from −0.5 to 0.5. The normalized *z*-scores are displayed on a color scale, ranging from blue (geometric means below the geometric mean of the total group) to red (geometric means above the geometric mean of the total group). The white color indicates values equal to the group geometric mean. The stronger the deviation from the group geometric mean, the darker the color. Comparison of the CD4 naïve cells **(B)**, CD4 TemRA cells **(C)**, B-cells **(D)**, naïve B-cells **(E)**, and the CD4/CD8 ratio **(F)** TruCOUNT kinetics between the participants with low or high pre-vaccination CMI. The groups were compared per subset with the Mann–Whitney *U* test. *p < 0.05 and **p < 0.01.

Finally, we determined whether the blood leukocyte responses were different between participants with low and high pre-CMI (Figure S5 in Supplementary Material; Figure 5A). Pre-vaccination, a significant difference was found in the CD4/CD8 T-cell ratio between the two groups: participants with low pre-CMI in general possessed a CD4/CD8 ratio above 3, whereas participants with high pre-CMI showed a ratio below 3. This possibly suggests a compositional difference in the immune phenotype between participants with low and high pre-CMI. Participants with low pre- CMI showed slightly higher numbers of CD4 T-cells (mainly naïve cells), whereas participants with high pre-CMI showed slightly higher numbers of CD8 T-cells. This difference in CD4/CD8 T-cell ratio continued to exist at the different time points after vaccination (Figures 5A,F). Correspondingly, at 14 days post-vaccination, significantly higher numbers of circulating CD4 naïve cells, CD4 TemRA cells, B-cells, and naïve mature B-cells were found in the participants with low pre-CMI (Figures 5A–E). This result indicates differential lymphocyte subset kinetics in participants with low and high pre-CMI 14 days post-vaccination and therefore suggests that these responses can be used as a short-term biomarker to predict the long-term vaccine responses in middle-aged adults.

## Discussion

Our study showed that the pre-vaccination VZV-specific cellular immunity highly affects the VZV vaccine-induced immune responses in middle-aged adults. Robust long-term increases in VZV-specific CMI, here defined as the numbers of IFNγ-producing cells, were observed post-vaccination in participants with low pre-CMI, whereas no significant enhancement was observed in participants with high pre-CMI. Participants with low pre-CMI mainly showed long-term enhancement of activated (based on the double expression of CD38 and HLA-DR) CD4 T-cells, while participants with high pre-CMI demonstrated increased proportions of activated VZV-specific CD8 TemRA cells, suggesting exhaustion of the VZV-specific T-cell response. Interestingly, the middle-aged participants with low pre-CMI were generally older than participants with high pre-CMI and had a higher CD4/CD8 T-cell ratio (>3). Based on these findings, we propose that next to age, pre-vaccination VZV-specific CMI and a high CD4/CD8 T-cell ratio (>3) are predictive for a productive immune response to VZV vaccination at middle age. Moreover, whole blood kinetics, as found in the absolute numbers of CD4 naïve cells, CD4 TemRA cells, B-cells, and naïve mature B-cells 14 days post-vaccination, might be used as a quick short-term predictive parameter for the long-term enhancement of VZV-specific CMI.

Previously, a large study (*N* = 22,439) in Northern America and Europe estimated the efficacy of the Zostavax vaccination at 50–59 years of age to be approximately 70% (29). This efficacy sharply declined toward older age groups (10, 19), suggesting possible benefits of timely vaccination to boost the VZV-specific CMI before reaching old age. Since childhood varicella vaccination has been implemented in the national immunization program of several countries across Europe as well as the US, the VZV-specific epidemiology in these countries significantly differs from those not vaccinating against VZV (5, 16, 30). Consequently, the immune responses to the Zostavax vaccine in middle-aged adults as investigated in countries with childhood VZV vaccination should not be directly extrapolated to our study population, in which VZV-specific CMI is more frequently boosted by external natural virus circulation. Despite these epidemiological differences, our results show similar short-term enhancement of the VZV-specific CMI in the circulation of middle-aged adults as was observed previously (31).

Despite high variation in vaccine responses, the differential peripheral responses in participants with low and high pre-CMI are remarkable and suggest that mainly middle-aged adults with low pre-vaccination CMI benefit from additional immunization. Benefits of the vaccination might not be excluded in participants with high pre-CMI, since memory immunity locally in lymph nodes and tissue could be increased. Interestingly, more participants in their early fifties were found in the high pre-CMI group, which might indicate regular natural boosting of VZV in this age group. As a consequence of waning immunity to natural infection in combination with immunosenescence (10), the effect of pre-vaccination immunity might be diminished in older age groups. Therefore, the optimum age for VZV vaccination might be better defined by balancing the combined effects of pre-vaccination immunity and age. Based on our findings, although numbers of participants were low, we speculate that VZV vaccination might be beneficial for participants in their sixties, when pre-vaccination levels are relatively low and effective vaccine responses are obtained.

Remarkably, the long-term enhancement of the terminally differentiated CD8 TemRA cells in participants with high pre-CMI may suggest exhaustion of the VZV-specific T-cell pool, although a possibly beneficial effect on the function of these CD8 TemRA cells in prevention of viral replication cannot be excluded. Although the exhaustion needs to be confirmed by measurement of several exhaustion markers (32), our results might indicate that an additional VZV vaccination in participants with already high levels of pre-vaccination VZV-specific CMI may unintentionally cause exhaustion of the VZV-specific T-cell pool. This finding may be the result of repeatedly stimulation of VZV-specific T-cells by frequent natural boosting in these participants. Of note, the vaccination did not induce elevated IL10 production or elevated numbers of circulating Treg cells in the participants with high pre-CMI, suggesting that the vaccine did not affect the suppression of the immune response in these participants.

This indication of these potentially large effects of the pre-vaccination VZV-specific CMI on the VZV-specific vaccine response requires additional investigation to improve our understanding of VZV-specific cellular immune responses. Consequently the implementation of a vaccination strategy might enhance the protection of older adults against herpes zoster disease. Since the pre-vaccination immunity in middle-aged adults may be different between countries with and without childhood varicella vaccination, it is of interest to compare the cellular immune responses obtained from this study, with that in middle-aged adults from a country with high childhood VZV-vaccination coverage. Importantly, a herpes zoster adjuvanted subunit vaccine containing the recombinant glycoprotein E is under evaluation for registration, after showing an efficacy rate of 97.2% in protection against herpes zoster disease of the elderly (20–22). To further improve our understanding of factors affecting VZV-specific immune responses, it would also be of interest to investigate the effects of pre-vaccination immunity on the T-cell responses initiated by this vaccine.

In addition, we are the first reporting an association between the CD4/CD8 T-cell ratio and VZV-specific CMI. Interestingly, we observed a significantly higher CD4/CD8 T-cell ratio (>3) in participants with low pre-vaccination CMI, suggesting a difference in the immune phenotype between the two groups. Considering the significantly higher age of the participants with low pre-CMI in our study population, our results confirm and extend the observation of a general increase in the CD4/CD8 T-cell ratio with advancing age, as previously shown by others (33). The higher CD4/CD8 T-cell ratio, above 3, perhaps indicates immune competence in participants with low pre-vaccination VZV-specific CMI, and thereby predicts which participants are eligible for timely VZV vaccination. Larger cohort studies are needed to confirm this observation. Of note, we did not observe a negative correlation between the numbers of Treg cells or late-differentiated CD4 T-cells and the VZV vaccine response, as was previously found in nursing home elderly (34). This difference might be explained by the low number of participants in our study.

Our study is limited by the measurement of VZV-specific CMI in the circulation only, which is likely to have a different cellular composition compared with tissue sites (28, 35). For example, a distinct effect of age was found on peripheral T-cells as compared with other compartments (35). Specifically, in contrast to an age related decrease in VZV-specific CD4 T-cell numbers in the circulation, no age related decrease was found in the skin (15). In our study, it cannot be excluded that participants with high circulatory levels of VZV-specific CMI might show enhancement of VZV-specific CD4 T-cells in the skin, and therefore benefit more from the vaccination than concluded based on the analysis of the peripheral immune response. Moreover, the long-term immunogenicity of this study has to be confirmed by additional sampling of the participants at older age, years after vaccination, in a comparative study with elderly persons who did not receive an additional vaccination.

In conclusion, we found long-term beneficial effects of VZV (Zostavax) vaccination on the VZV-specific CMI in the circulation of middle-aged adults with low pre-vaccination CMI. Moreover, age (>60 years) and a high CD4/CD8 T-cell ratio (>3) were found predictive for the presence of low VZV-specific pre-CMI. More studies in independent cohorts are required to substantiate these findings. Our results suggest that in a population with high levels of natural VZV boosting, vaccination against herpes zoster is more beneficial in the early sixties and thereby adds important knowledge for the further development of strategies to prevent the high herpes zoster disease burden in the aging population.

## Ethics Statement

Written informed consent was obtained from all participants before the study. All procedures were in accordance with the Declaration of Helsinki. The study was approved by the Medical Research Ethics Committees United (Mec-U) in Nieuwegein, the Netherlands (NTR4636).

## Author Contributions

MH, MZ, GB, AB, and A-MB designed the study protocol. MH and LR planned and performed the clinical work and performed the laboratory experiments. MH, LR, MZ, AB, and A-MB analyzed and interpreted the data. MH, LR, MZ, GB, AB, and A-MB wrote and critically revised the manuscript.

## Conflict of Interest Statement

MH, LR, GB, and A-MB declare no conflict of interest. MZ reports grants from Erasmus MC, Rotterdam (The Netherlands) during the conduct of the study. AB is a consultant for Grunenthal GmbH (Germany).
