# Supplementary material for: Age-Dependent Pre-Vaccination Immunity Affects the Immunogenicity of Varicella Zoster Vaccination in Middle-aged Adults
Source: Front Immunol. 2018 Jan 23;9:46. doi: 10.3389/fimmu.2018.00046 (PMC5787056; doi:10.3389/fimmu.2018.00046)
Supplement: Supplementary file 1 [file Table_1.PDF]

Supplementary Table 1. Participant characteristics

medication used more than 3 months ago, N=51

**Parameter**	
BMI (range)	26.0 (20.3 – 38.9)
CMV seropositive (number, %)	22 (43.1%)
**Diseases in last year (number, %)**	
Diabetes type II	1 (2%)
High blood pressure	4 (7.8%)
Vascular diseases	3 (5.9%)
Lung diseases	2 (3.9%)
Rheumatic diseases	0 (0%)
Gastro-intestinal diseases	0 (0%)
Other diseases	3 (5.9%)
No serious diseases	41 (80.4%)
**Medication last 6 months (number, %)**	
Medication for infections #	5 (9.8%)
Cholesterol lowering medication	7 (13.7%)
Diabetic medication	0 (0%)
Blood pressure lowering medication	11 (21.6%)
Immunosuppressive medication #	1 (2%)
No medication	35 (68.6%)
**Infections (number, %)**	
Influenza < 4 weeks	7 (13.7%)
Cold <4 weeks	7 (13.7%)
No infection < 4 weeks	37 (72.5%)
**Smoking (number, %)**	
Cigarette smoking	6 (11.8%)
Pipe smoking	1 (2%)
No smoking	44 (86.3%)
**Previous episode of chickenpox (numbers, %)**	
Yes, at young age	32 (62.7%)
Yes, at older age	1 (2%)
No	1 (2%)
Don’t know	17 (33.3%)
**Recent contact with children with chickenpox (numbers, %)**	
Yes	3 (5.9%)
No	42 (82.4%)
Unknown	6 (11.8%)
