# Supplementary material for: Age-Dependent Pre-Vaccination Immunity Affects the Immunogenicity of Varicella Zoster Vaccination in Middle-aged Adults
Source: Front Immunol. 2018 Jan 23;9:46. doi: 10.3389/fimmu.2018.00046 (PMC5787056; doi:10.3389/fimmu.2018.00046)
Supplement: Supplementary file 2 [file Table_2.PDF]

Supplementary Table 2. Phenotypical definitions of the immune cells subsets

Subset	Phenotype definition
Monocytes	SSC^{inter}CD45+
Granulocytes	SSC^{high}CD45+
Lymphocytes	SSC^{low}CD45+
B-cells	SSC^{low}CD45+CD19+
Translational	SSC^{low}CD45+CD27-CD38+
Plasma cells	SSC^{low}CD45+CD27+CD38+
Naïve mature	SSC^{low}CD45+CD27-IgD+$CD38^{dim}$
Natural effector	SSC^{low}CD45+CD27+IgD+$CD38^{dim}$
CD27- memory	SSC^{low}CD45+CD27-IgD-$CD38^{dim}$
CD27+ memory	SSC^{low}CD45+CD27+IgD-$CD38^{dim}$
T-cells	SSC^{low}CD45+CD3+
CD4 T-cells	SSC^{low}CD45+CD3+CD4+
Tregs	SSC^{low}CD45+CD3+CD4+CD25+
CD4 naïve	SSC^{low}CD45+CD3+CD4+CD45RO-CCR7+
CD4 CM	SSC^{low}CD45+CD3+CD4+CD45RO+CCR7+
CD4 TemRA	SSC^{low}CD45+CD3+CD4+CD45RO-CCR7-
CD4 TemRO	SSC^{low}CD45+CD3+CD4+CD45RO+CCR7-
CD4CXCR5	SSC^{low}CD45+CD3+CD4+Cd45RO+CXCR5+
CD8 T-cells	SSC^{low}CD45+CD3+CD8+
CD8 naïve	SSC^{low}CD45+CD3+CD8+CD45RO-CCR7+
CD8 CM	SSC^{low}CD45+CD3+CD8+CD45RO+CCR7+
CD8 TemRA	SSC^{low}CD45+CD3+CD8+CD45RO-CCR7-
CD8 TemRO	SSC^{low}CD45+CD3+CD8+CD45RO+CCR7-
