# Supplementary material for: Age-Dependent Pre-Vaccination Immunity Affects the Immunogenicity of Varicella Zoster Vaccination in Middle-aged Adults
Source: Front Immunol. 2018 Jan 23;9:46. doi: 10.3389/fimmu.2018.00046 (PMC5787056; doi:10.3389/fimmu.2018.00046)
Supplement: Supplementary file 3 [file Image_1.PDF]

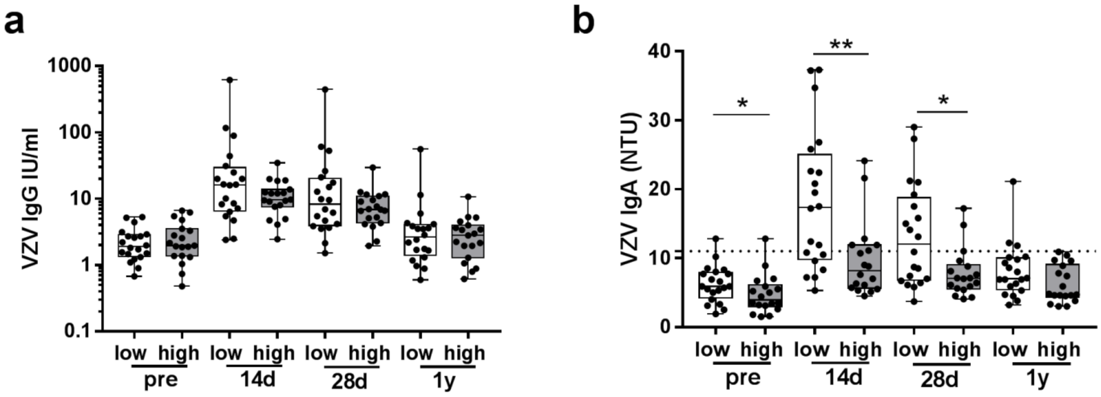

Supplementary Figure 1. VZV-specific antibody levels pre- and post-vaccination in participants with low and high pre-vaccination IFNy producing cells.

The IgG **(a)** and IgA **(b)** levels in participants with low (white boxplots) and high (grey boxplots) pre-CMI. All boxplots are plotted from the min to max values with indication of the median. The geometric mean concentrations with 95% CI were indicated in the graphs. The participants with low and high pre-CMI were compared with the Mann Whitney U test. *p<0.05, **p<0.01.
