# Supplementary material for: Age-Dependent Pre-Vaccination Immunity Affects the Immunogenicity of Varicella Zoster Vaccination in Middle-aged Adults
Source: Front Immunol. 2018 Jan 23;9:46. doi: 10.3389/fimmu.2018.00046 (PMC5787056; doi:10.3389/fimmu.2018.00046)
Supplement: Supplementary file 4 [file Image_2.PDF]

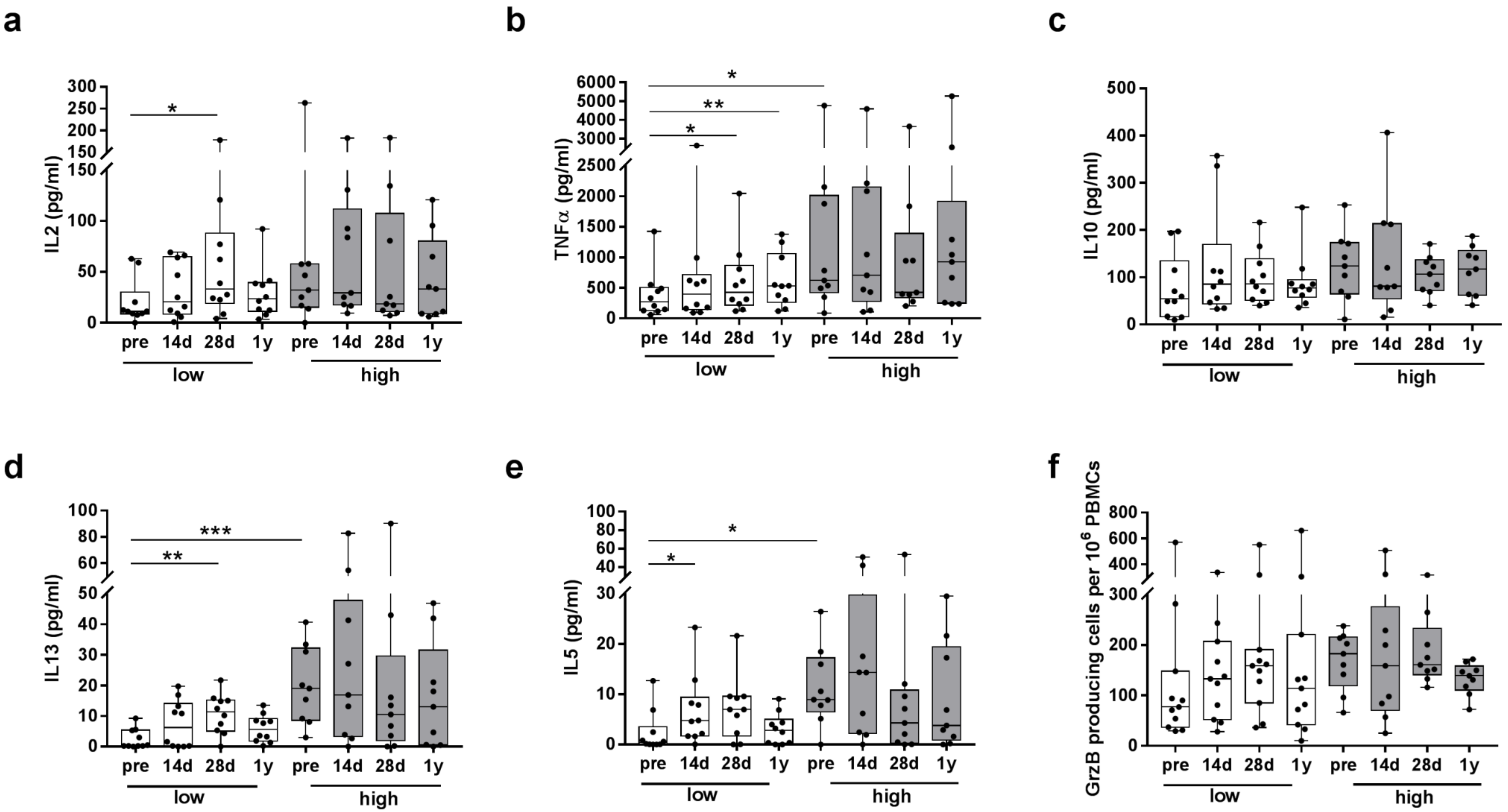

Supplementary figure 2. VZV-specific cytokine secretion in cell culture supernatant from participants with low and high pre-CMI.
Concentrations (pg/ml) of VZV-specific IL2 **(a)**, TNFα **(b)**, IL10 **(c)**, IL13 **(d)**, and IL5 **(e)** in cell culture supernatant after 48h of stimulation with VZ10 in participant with low (white boxplots) N=10 and high (grey boxplots) N=9 pre-CMI. **(f)** The number of GrzB producing cells per 10^6 PBMCs pre- and post-vaccination in participants with low (white boxes) N=11 and high (grey boxes) N=9 pre-CMI as measured by the Elispot assay. The bars were plotted from the min to the max values, with the middle-line indicating the median. The participants with low and high pre-CMI were compared with the Mann Whithney U test at all different time points. The different time points were compared with the Wilcoxon signed rank test preceded by the Friedman test for the participants with low and high pre-CMI separately. *p<0.05, **p<0.01, ***p<0.001.
