# Supplementary figures and images for: Age-Dependent Pre-Vaccination Immunity Affects the Immunogenicity of Varicella Zoster Vaccination in Middle-aged Adults
Source: Front Immunol. 2018 Jan 23;9:46. doi: 10.3389/fimmu.2018.00046 (PMC5787056; doi:10.3389/fimmu.2018.00046)

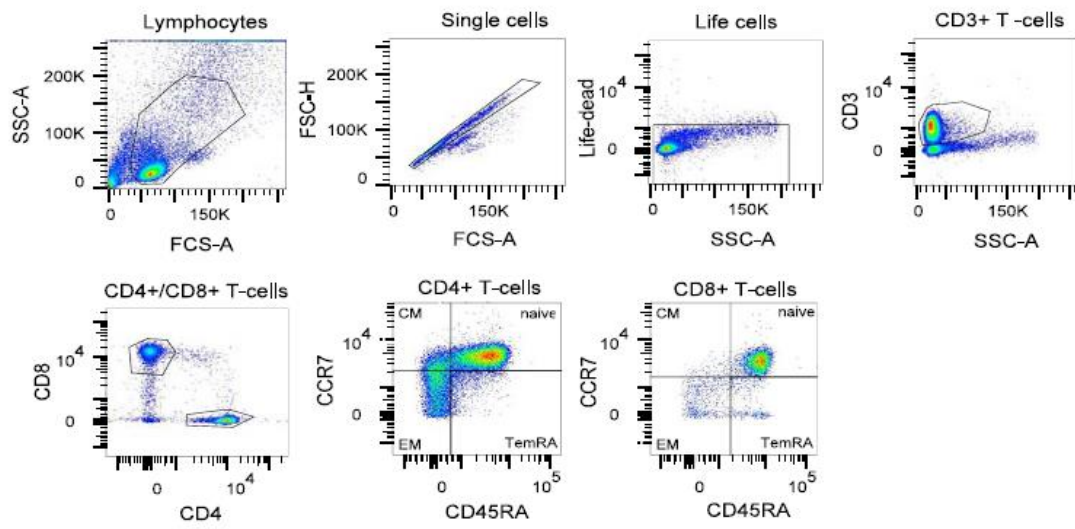

**Supplementary Figure 3. Gating strategy**

Supplement: Supplementary file 5 [file Image_3.PDF]
