# Supplementary material for: Age-Dependent Pre-Vaccination Immunity Affects the Immunogenicity of Varicella Zoster Vaccination in Middle-aged Adults
Source: Front Immunol. 2018 Jan 23;9:46. doi: 10.3389/fimmu.2018.00046 (PMC5787056; doi:10.3389/fimmu.2018.00046)
Supplement: Supplementary file 6 [file Image_4.PDF]

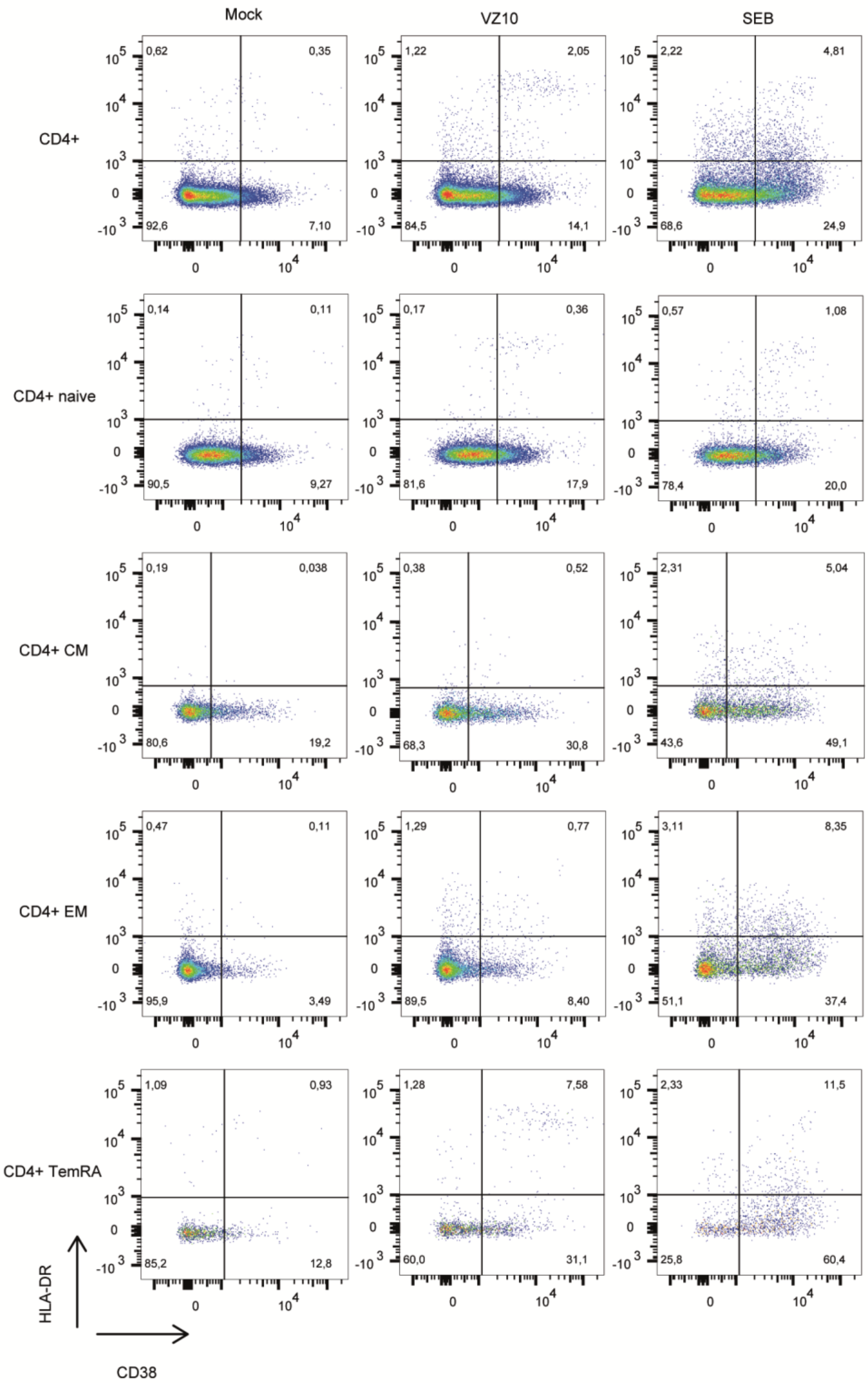

Supplementary Figure 3. Gating strategy of the activated CD4 T-cell subsets.
The VZ10 specific activated cells (based on the double expression of CD38 and HLA-DR) in the different CD4 T-cell subsets representative to the negative mock control and the positive (SEB) control from a representative sample 14 days post-vaccination.
