# Supplementary material for: Age-Dependent Pre-Vaccination Immunity Affects the Immunogenicity of Varicella Zoster Vaccination in Middle-aged Adults
Source: Front Immunol. 2018 Jan 23;9:46. doi: 10.3389/fimmu.2018.00046 (PMC5787056; doi:10.3389/fimmu.2018.00046)
Supplement: Supplementary file 7 [file Image_5.PDF]

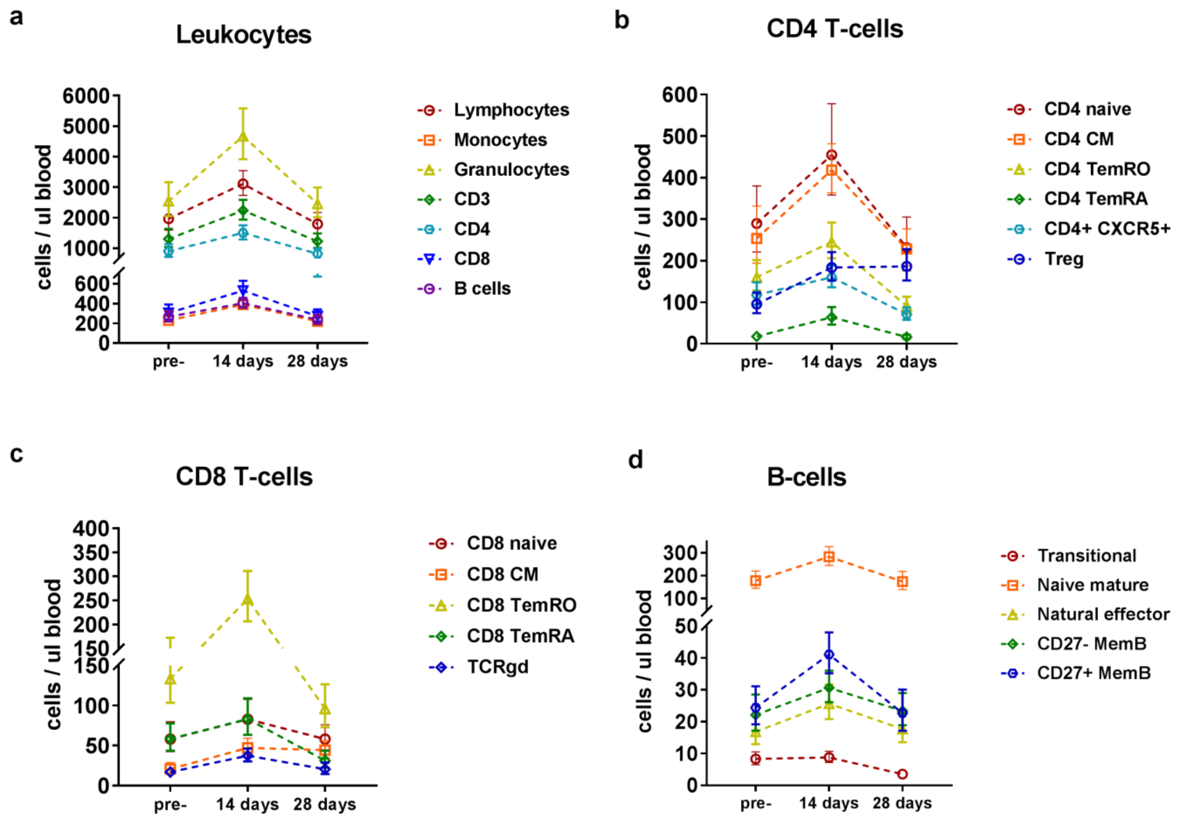

Supplementary Figure 4. Leukocyte subset numbers pre- and post- VZV vaccination in middle-aged adults. Absolute numbers of leukocyte subsets **(a)**, CD4 T-cell subsets **(b)**, CD8 T-cell subsets **(c)**, and B-cell subsets **(d)** pre- and 14 days, and 28 days post-vaccination, N = 49. **(a)** The absolute numbers of all subsets were significantly elevated 14 days post-vaccination (lymphocytes: ρ= 0.0001, monocytes: ρ= 0.0002, granulocytes: ρ= 0.0025, CD3: ρ=< 0.0001, CD4: ρ=0.0002, CD8: ρ<0.0001, B-cells: ρ=0.0006), and were reduced to baseline levels again after 28 days. **(b)** The absolute numbers of all subsets were significantly elevated 14 days post-vaccination (CD4 naive: ρ=0.009, CD4 CM: ρ<0.0001, CD4 TemRO: ρ=0.007, CD4 TemRA: ρ<0.0001, CD4+CXCR5+: ρ=0.007, Treg: ρ<0.0001). After 28 days, the absolute numbers of CD4 TemRO (ρ=0.02) and CD4+CXCR5+ (ρ=0.007) were significantly lowered compared to baseline levels. Treg (ρ<0.0001) cell numbers were significantly elevated after 28 days. **(c).** The absolute numbers of all subsets were significantly elevated after 14 days (CD8 naïve: ρ=0.0002, CD8 CM: ρ<0.0001, CD8 TemRO: ρ=0.0002, CD8 TemRA: ρ=0.05, TCRgd: ρ<0.0001). The absolute numbers of CD8 CM cells were significantly elevated after 28 days (ρ=0.002), and the absolute numbers of CD8 TemRA cells significantly decreased (ρ=0.004). **(d).** The absolute numbers of naive mature (ρ=0.008), natural effector (ρ=0.004), CD27-MemB (ρ= 0.003), and CD27+MemB (ρ<0.0001) were significantly elevated after 14 days. The absolute numbers of transitional cells were significantly decreased after 28 days (ρ<0.0001). The absolute numbers at the 14 days and 28 days post-vaccination time points were compared to the pre-vaccination levels with the Wilxocon signed rank test preceded by the Friedman test and were corrected for multiple comparisons.
